# Volatile Metabolites in Lavage Fluid Are Correlated with Cytokine Production in a Valley Fever Murine Model

**DOI:** 10.3390/jof9010115

**Published:** 2023-01-14

**Authors:** Emily A. Higgins Keppler, Marley C. Caballero Van Dyke, Heather L. Mead, Douglas F. Lake, D. Mitchell Magee, Bridget M. Barker, Heather D. Bean

**Affiliations:** 1 School of Life Sciences, Arizona State University, Tempe, AZ 85287, USA; 2 Center for Fundamental and Applied Microbiomics, The Biodesign Institute, Arizona State University, Tempe, AZ 85287, USA; 3 Pathogen and Microbiome Institute, Northern Arizona University, Flagstaff, AZ 86011, USA; 4 Center for Personalized Diagnostics, The Biodesign Institute, Arizona State University, Tempe, AZ 85287, USA

**Keywords:** Valley fever, coccidioidomycosis, fungal pneumonia, mouse model, biomarkers, comprehensive two-dimensional gas chromatography, GC×GC, untargeted metabolomics

## Abstract

*Coccidioides immitis* and *Coccidioides posadasii* are soil-dwelling fungi of arid regions in North and South America that are responsible for Valley fever (coccidioidomycosis). Forty percent of patients with Valley fever exhibit symptoms ranging from mild, self-limiting respiratory infections to severe, life-threatening pneumonia that requires treatment. Misdiagnosis as bacterial pneumonia commonly occurs in symptomatic Valley fever cases, resulting in inappropriate treatment with antibiotics, increased medical costs, and delay in diagnosis. In this proof-of-concept study, we explored the feasibility of developing breath-based diagnostics for Valley fever using a murine lung infection model. To investigate potential volatile biomarkers of Valley fever that arise from host–pathogen interactions, we infected C57BL/6J mice with *C. immitis* RS (*n* = 6), *C. posadasii* Silveira (*n* = 6), or phosphate-buffered saline (*n* = 4) via intranasal inoculation. We measured fungal dissemination and collected bronchoalveolar lavage fluid (BALF) for cytokine profiling and for untargeted volatile metabolomics via solid-phase microextraction (SPME) and two-dimensional gas chromatography coupled with time-of-flight mass spectrometry (GC×GC-TOFMS). We identified 36 volatile organic compounds (VOCs) that were significantly correlated (*p* < 0.05) with cytokine abundance. These 36 VOCs clustered mice by their cytokine production and were also able to separate mice with moderate-to-high cytokine production by infection strain. The data presented here show that *Coccidioides* and/or the host produce volatile metabolites that may yield biomarkers for a Valley fever breath test that can detect coccidioidal infection and provide clinically relevant information on primary pulmonary disease severity.

## 1. Introduction

*Coccidioides immitis* and *C. posadasii* are soil-dwelling fungi of the arid regions of North and South America that cause the fungal infection coccidioidomycosis, or Valley fever, when arthroconidia are inhaled. One-half to two-thirds of individuals infected with *Coccidioides* remain asymptomatic or exhibit mild symptoms and resolve their clinical disease without seeking medical attention, retaining long-lived immunity [1]. Symptoms develop in approximately forty percent of Valley fever cases, ranging from mild, self-limiting respiratory infections to severe, life-threatening pneumonia, and a small percentage will result in disseminated disease [2]. The most common diagnosis is acute or subacute pneumonic illness, which usually does not require treatment; instead, patients are monitored until the infection is resolved on its own [1]. Conversely, patients with extensive spread of infection or who are at high risk of complications due to immunosuppression or other preexisting conditions require a variety of treatment strategies, including antifungal therapy, surgical debridement, or both [1].

Misdiagnosis occurs frequently in symptomatic Valley fever. Without testing, it is often mistaken for a bacterial pneumonia and inappropriately treated with antibiotics. The only conclusive diagnosis of Valley fever requires a positive fungal culture or histopathology showing spherules or endospores within lung tissue [3]. However, due to the combination of invasiveness, time, and expense of histopathological or culture-based diagnostics, most patients are diagnosed through serological testing, but low sensitivities contribute to diagnostic delays. Enzyme immunoassay (EIA), immunodiffusion (ID), and complement fixation (CF) tests are all commercially available; however, EIA tests for IgG and IgM range in sensitivity from 47% to 87% and 22% to 61%, respectively, and ID and CF tests are between 60% and 91% and 65% and 98% sensitive, respectively [2]. The combination of insufficient testing for fungal pneumonias and insufficient sensitivities in the existing diagnostics led to more than a month’s delay in diagnosis for 46% of the Valley fever patients in the endemic region of Phoenix, Arizona, United States, significantly increasing medical costs [4]. Surveillance in nonendemic states showed that 70% of Valley fever patients were diagnosed with another condition before being tested for coccidioidomycosis, with a median delay in diagnosis of 38 days [5].

Breath-based diagnostics are increasingly being pursued as a means of diagnosing respiratory infections. Animal model respiratory infections have shown that the combination of pathogen and host volatile metabolites have high diagnostic accuracy for detecting and identifying lung infection etiology [6,7,8,9,10,11,12,13,14,15]. Further, clinical studies of community-acquired pneumonia, ventilator-associated pneumonia, and chronic lung infections have shown that volatile biomarkers can sensitively detect and identify the bacterial and fungal etiologies through the analysis of sputum, bronchoalveolar lavage fluid (BALF), and breath volatiles [16,17,18,19]. In this proof-of-concept study, we explored the feasibility of developing breath-based diagnostics for Valley fever using a murine lung infection model. To demonstrate that volatile *Coccidioides* biomarkers can be detected in vivo and to identify volatile biomarkers of Valley fever that arise from host–pathogen interactions, we inoculated 16 mice with *C. immitis* (*n* = 6), *C. posadasii* (*n* = 6), or phosphate-buffered saline (*n* = 4) and collected BALF for untargeted volatile metabolomics via two-dimensional gas chromatography coupled with time-of-flight mass spectrometry (GC×GC-TOFMS). Since an antibody response is often delayed, we hypothesized that *Coccidioides* and the host would produce volatile metabolites in response to infection that can be utilized as sensitive and specific biomarkers for Valley fever infections.

## 2. Methods and Materials

### 2.1. Mice

Female C57BL/6J mice (Jackson Laboratory, Bar Harbor, ME, USA) 6–8 weeks of age were used for these studies. Mice were housed according to NIH guidelines for housing and care in a biosafety level 3 animal laboratory. All procedures were approved by the Institutional Animal Care and Use Committee (protocol 16–011) of Northern Arizona University.

### 2.2. Pulmonary Coccidioidal Infections

The *Coccidioides* isolates used in this study were the type strains *C. immitis* strain RS (ATCC^®^ catalog no. NR-48942; NCBI accession no. AAEC00000000.3) and *C. posadasii* strain Silveira (ATCC^®^ catalog no. NR-48944; NCBI accession no. ABAI00000000.2). Mice were anesthetized with ketamine/xylene (80/8 mg/kg) and intranasally inoculated with 100 arthroconidia of *C. immitis* strain RS (*n* = 6) or *C. posadasii* strain Silveira (*n* = 6) suspended in 30 μL phosphate-buffered saline (PBS), as described previously [20,21]. Control mice were inoculated with PBS alone (*n* = 4). The mice were killed at day 10 postinoculation. The lungs were rinsed with 2 mL of PBS to collect bronchoalveolar lavage fluid (BALF), which was filtered with 0.22 μm Ultrafree^®^-MC centrifugal filter devices with Durapore^®^ membrane (MilliporeSigma, Burlington, MA, USA). One milliliter of each BALF sample was stored at −80 °C for volatilomic analysis. Halt™ Protease Inhibitor Cocktail (10 μL/mL; ThermoFisher Scientific, Waltham, MA, USA) was added to the remainder of each BALF sample for cytokine analysis. Spleens and brains were homogenized in 1 mL of sterile PBS followed by culture of 10-fold dilutions of each tissue on 2X GYE agar (2% glucose (VWR™, Radnor, PA, USA), 1% yeast extract (BD™, Franklin Lakes, NJ, USA, and 1.5% bacteriological agar (BD Difco, Franklin Lakes, NJ, USA)) to assess fungal dissemination.

### 2.3. Cytokine Analysis

Cytokine levels in the BALF samples were examined using the cytokine mouse magnetic Invitrogen™ 26-Plex ProcartaPlex™ Panel 1 (25 μL of sample; ThermoFisher Scientific) per manufacturer’s instructions using a 2 h incubation. Samples were read using a MAGPIX^®^ multiplex system (Luminex^®^ Corporation, Austin, TX, USA) with Luminex^®^ xPONENT^®^ 3.0 software.

### 2.4. Volatile Metabolomic Analysis by SPME-GC×GC-TOFMS

The BALF samples were allowed to thaw at 4 °C overnight, and then split into technical triplicates of 200 μL that were transferred and sealed into sterilized 2 mL GC headspace vials with Supelco^®^ PTFE/silicone septum magnetic screw caps (Sigma-Aldrich^®^, St. Louis, MO, USA). Samples were randomized for analysis. Volatile metabolites sampling was performed by solid-phase microextraction (SPME) using a Gerstel^®^ MPS Robotic Pro MultiPurpose autosampler directed by Maestro^®^ software (Gerstel^®^, Inc., Linthicum, MD, USA). Sample extraction and injection parameters are provided in Table S1 (see Autosampler Method). Volatile metabolite analysis was performed by two-dimensional gas chromatography−time-of-flight mass spectrometry (GC×GC–TOFMS) using a LECO^®^ Pegasus^®^ 4D and Agilent^®^ 7890B GC (LECO^®^ Corp., St. Joseph, MI, USA). Chromatographic, mass spectrometric, and peak detection parameters are provided in Table S1 (see GC×GC Method and Mass Spectrometry Method). An external alkane standards mixture (C_8_–C_20_; Sigma-Aldrich^®^) was sampled multiple times for calculating retention indices (RI). The injection, chromatographic, and mass spectrometric methods for analyzing the alkane standards were the same as for the samples.

### 2.5. Processing and Analysis of Chromatographic Data

Data collection, processing, and alignment were performed using ChromaTOF software version 4.71 with the Statistical Compare package (LECO^®^ Corp.), using the parameters listed in Table S1 (see Data Processing Method). Peaks were assigned a putative identification based on mass spectral similarity and retention index (RI) data, and the confidence of those identifications are indicated by assigning levels 1 to 4 (1 highest) [22]. Peaks at level 1 were identified based on mass spectral and RI matches with external standards. Peaks at level 2 were identified based on ≥800 mass spectral matches by a forward and reverse search of the NIST 14 library and RIs that were consistent (<7% error) with a calculated 624 RIs using the following equation: mean NIST nonpolar RI × 1.0517 + 14.468. This equation was obtained based on the linear relationship of RIs observed between a 624Sil column and nonpolar stationary phase [23]. Level 1, 2, and 3 compounds were assigned to chemical functional groups based upon characteristic mass spectral fragmentation patterns and second-dimension retention times, as previously described [24]. Level 4 compounds have mass spectral matches <600 or RIs that do not match previously published values and are reported as unknowns.

### 2.6. Data Postprocessing and Statistical Analyses

#### 2.6.1. Cytokine Data

Principal component analysis was performed using *prcomp* in R *stats* package version 4.1.2 with the inoculated mice (*n* = 12) and PBS mice (*n* = 4) as observations and the log_10_ transformed cytokine abundance (mean-centered and scaled to unit variance) as variables. The relatedness of mice based on their cytokine profiles were assessed using hierarchical clustering analysis on the Euclidean distance between mice and cytokines with average linkage using R *pheatmap* package version 1.0.12.

#### 2.6.2. Volatile Data

The data postprocessing steps are depicted in Figure S1. Before statistical analyses, compounds eluting prior to 358 s (acetone retention time) and siloxanes (i.e., chromatographic artifacts) were removed from the peak table. Missing values were handled using RepHM imputation in the R *MetabImpute* package version 0.1.0, as follows: peaks that were present in only one of the three technical replicates were imputed to zero for that replicate, while missing values for peaks that were present in two out of three technical replicates were imputed to half of the minimum value within the replicates (as described in [25]). The relative abundance of compounds across chromatograms was normalized using probabilistic quotient normalization (PQN) [26] in R version 4.1.2. The data were log_10_ transformed, and geometric means of the technical triplicates were calculated. Principal component analyses were performed using *prcomp* in R *stats* package version 4.1.2 with the geometric means of the technical replicates as observations and the absolute peak intensities (mean-centered and scaled to unit variance) as variables. Kendall correlation analysis was performed correlating peak intensities with cytokine abundance. Analytes with absolute values of Kendall correlation scores of |τ| > 0.3 and *p* < 0.05 were considered to be significantly correlated. The relatedness of samples based on their volatile metabolomes were assessed using hierarchical clustering analysis in R *pheatmap* package version 1.0.12, using Pearson correlations with average linkage.

## 3. Results

### 3.1. Coccidioides-Infected C57BL/6J Mice Exhibit a Gradient of Cytokine Production

**Figure 1:**
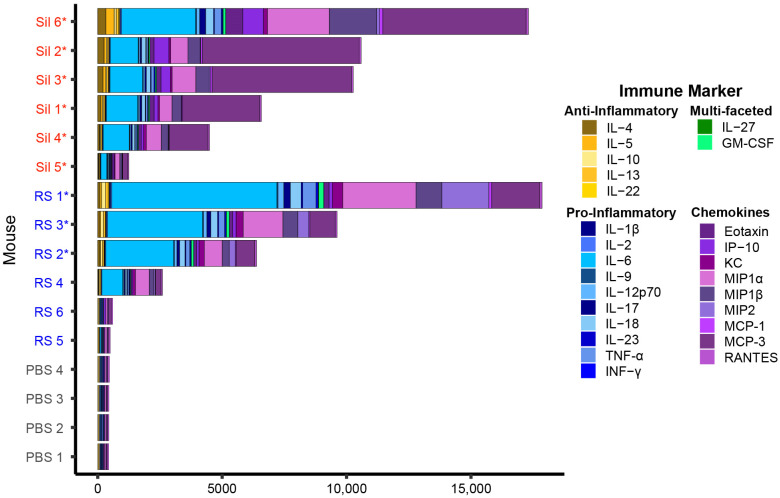
There is a gradient of total cytokine concentrations in the BALF of *Coccidioides*-inoculated mice. A total of 26 cytokines are shown for individual mice inoculated with *C. immitis* strain RS (blue) and *C. posadasii* strain Silveira (Sil; red), or sham-inoculated with PBS (gray). Mice with disseminated disease are indicated with an asterisk (fungal counts in the spleen and brain are provided in Supplementary Table S2). Immune markers are color-coded by type, and should be read from left to right in the bar graph in order to match them with their labels in the legend, listed from top to bottom. Cytokine production was dominated by pro-inflammatory cytokines (blue) and chemokines (purple), while anti-inflammatory (yellow) and multifaceted (green) cytokines were produced at low levels.

We utilized a C57BL/6J murine infection model to identify lung volatiles that are correlated with Valley fever, and first measured bronchoalveolar lavage fluid (BALF) cytokines and fungal dissemination to characterize the degree of infection in each inoculated mouse. Six mice from each infection group, *Coccidioides immitis* strain RS and *C. posadasii* strain Silveira (Sil), and four control mice that were sham-infected with phosphate-buffered saline (PBS) were killed 10 days postinoculation. BALF was collected and analyzed for the abundance of 26 immune markers, and disseminated disease was assessed by quantifying the number of fungal colonies cultured from the mouse spleen and brain. All four PBS-inoculated mice produced very low levels of cytokines, but the Cocci-inoculated mice produced a wide range of immune responses to the fungus (Figure 1; Supplementary Table S2), which may indicate that the mice received varying doses of arthroconidia. The correlation between fungal dose and immune response in murine model lung infections of coccidioidomycosis has been previously reported [27,28]. Two of the Cocci-inoculated mice, RS 1 and Sil 6, produced a high abundance of cytokines: 39- and 38-fold greater than PBS mice, respectively. Two mice, RS 5 and RS 6, produced very low levels of cytokines, only 1.1- and 1.3-fold greater, respectively, than the abundance detected in the BALF of PBS mice, though the cytokine profiles of RS 5 and 6 were different from the uninfected controls, suggesting that they did receive at least a small dose of the fungus (Supplementary Figure S2). The remaining Cocci-inoculated mice produced a gradient of cytokine concentrations from 3- to 23-fold greater than controls. Generally, fungal dissemination correlated with the degree of cytokine response, independently of the cytokine profile. Fungus was recovered from the spleen and brain in the three RS mice with the highest cytokine responses and in the spleen of all six of the Silveira mice, four of which also had detectable dissemination to the brain (Figure 1, Supplementary Table S2).

**Figure 2:**
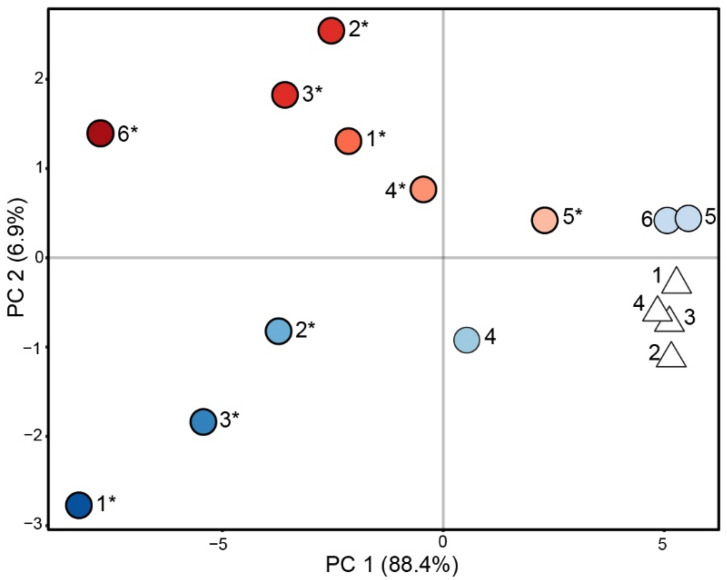
Differences in total cytokine production are the dominant source of variance driving separation of the mice in a Valley fever infection model. A principal component analysis (PCA) score plot of 16 mice inoculated with *C. immitis* RS (blue circles, *n* = 6), *C. posadasii* Silveira (red circles, *n* = 6) or PBS (white triangles, *n* = 4) as observations, using 26 cytokines as variables shows that total cytokine abundance separates the mice on PC1, representing the majority of the total variance. The color gradient in the observation markers indicates total cytokine abundance, with the darkest colors indicating the highest abundance; mice with disseminated disease are indicated with an asterisk (*). Differences in the cytokine profiles between RS and Sil, represented on PC2, are small in comparison.

Similarly to other studies characterizing the murine immune responses to RS and Silveira [21,29,30,31], we detected differences in the cytokine profiles of mice infected with these two strains, e.g., we found that IL-6 is more abundant in RS-infected mice and MCP-3 is more abundant in Silveira (Figure 1). However, the dominating pattern that we observe in the inoculated cohort data is based on the differences in total cytokine production. Using principal component analysis (PCA) to cluster the 12 Cocci-inoculated and 4 PBS mice based on similarities in their cytokine profiles, we observed that the mice separated predominantly by total cytokine concentrations on principal component 1 (PC1), which represents 88.4% of the total variance in the cytokine profiles. The two mice with the highest overall cytokine abundances are on the left, and the two mice with the lowest cytokine abundances, RS 5 and RS 6, are on the right clustering near the PBS controls on PC1 (Figure 2). Compared to the large amount of variance attributed to total cytokine abundances, the strain-level difference in the cytokine profiles is relatively small and reflected by the separation of the strains on PC2, which captures only 6.9% of the total variance. A hierarchical clustering analysis (HCA) of the mice based on their cytokine profiles generates two clusters, with one cluster containing the mice with moderate-to-high cytokine production and the other containing the PBS mice and the three Cocci-inoculated mice with the lowest cytokine production (Supplementary Figure S3). Within the moderate-to-high cytokine production cluster, there is not a clear separation in the mice by fungal strain; rather, the clustering is based upon overall cytokine abundance. Collectively, these data demonstrate that (1) there is not a clear delineation between the inoculated and control cohorts; rather, (2) there is a large gradient in the abundance of cytokines in response to fungal exposure that is the dominant source of variance in the infection models; and (3) the strain-specific differences we measured in the cytokine profiles of the inoculated mice are only a minor contributor to the overall variance observed in the model. All three of these factors influenced our interpretation of the volatiles data.

### 3.2. Murine Coccidioidomycosis Volatilome and Its Correlation with Cytokine Production

The volatile compounds present in the BALF samples were collected using solid-phase microextraction (SPME) and analyzed using two-dimensional gas chromatography coupled with time-of-flight mass spectrometry (GC×GC-TOFMS). After contaminant removal and data cleanup, we detected 91 VOCs (Supplemental Table S3), of which 26 were identified at level 1 or 2 [22] and assigned putative compound names based on mass spectral and chromatographic data (Table 1). Of the 26 named compounds, 19 have been previously associated with human and environmental fungal pathogens, including decanal, which was detected in the in vitro cultures of *Coccidioides* spp. [32] (Table 1). For the unnamed compounds, 16 were identified at level 3 and assigned chemical classification based on a combination of mass spectral and chromatographic data (Supplemental Table S3).

**Figure 3:**
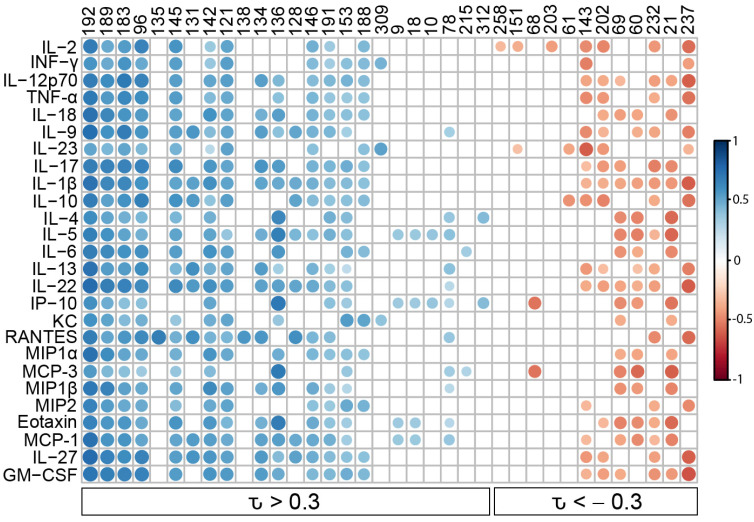
A subset of the volatilome is correlated to the cytokines in BALF. Kendall correlations were calculated between the VOCs and the cytokines in BALF of 12 Cocci-inoculated and 4 PBS-inoculated mice. This Kendall correlation plot represents the 36 volatile organic compounds (VOCs) (columns) that are significantly correlated with at least one of the 26 cytokines (rows). Circles indicate statistically significant correlations (*p* < 0.05), with positive correlations in blue (τ > 0.3), negative correlations in red (τ < −0.3), and darker colors and larger sizes indicating a stronger correlation. The volatiles are ordered by mean correlation from most positive (**left**) to most negative (**right**). Additional information about the VOCs, including putative identities, is provided in Supplementary Table S3.

To determine the relationship between *Coccidioides* infection and the BALF volatilome, we performed PCA using the 91 VOCs as variables and the mice as observations and detected a trend toward the mice separating on PC1 in a manner that reflected the pattern we observed for cytokine production (Supplementary Figure S4). This suggests that the volatiles are dependent, in part, on the immune response to fungal exposure. Using all 91 of the VOCs, however, only 18.3% of the volatilome variance is represented by PC1, and the separation of the mice by immune response was weaker than we observed for the cytokines, likely because only a subset of the BALF VOCs was altered by *Coccidioides* infection. A Kendall correlation between the 26 immune markers and 91 volatiles showed that 36 VOCs (40% of the total volatilome) were significantly correlated to at least one cytokine (|τ| > 0.3; *p* < 0.05; Figure S5 and Figure 3). As was seen in a previous study on the relationship between infection VOCs and immune response in murine model lung infections [10], here each VOC is either positively or negatively correlated with the full suite of cytokines, versus the VOCs having mixed patterns of positive and negative correlation. Therefore, the abundances of the immune-correlated VOCs are correlated with overall cytokine production, rather than to specific immune pathways.

**Figure 4:**
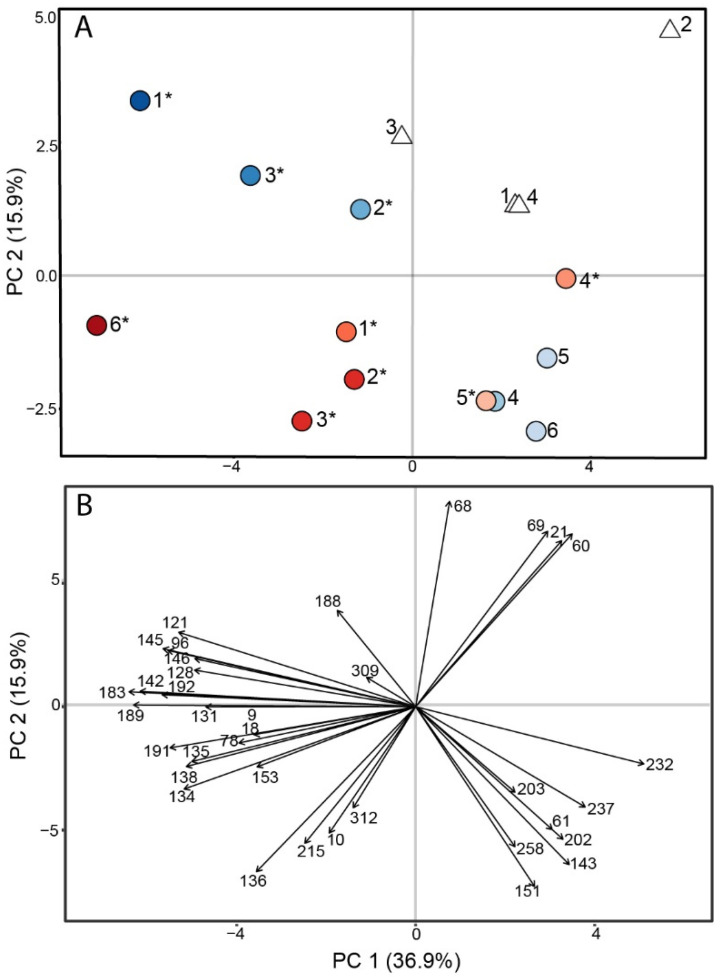
Immune-correlated VOCs recapitulate the clustering patterns produced by BALF cytokines. Principal component analysis score plot (**A**) and loading plot (**B**) using 36 immune-correlated volatile organic compounds (VOCs) from BALF as variables, and mice inoculated with *C. immitis* RS (blue circles), *C. posadasii* Silveira (red circles) or PBS (white triangles) as observations. The score plot (**A**) shows the mice separate on PC1 in a pattern that corresponds to their total cytokine production, and mice with moderate-to-high levels of cytokines separate by infection strain on PC2. The color gradient of the markers, darker to lighter, indicates total cytokine abundance, higher to lower; disseminated disease is indicated with an asterisk. The loading plot (**B**) shows the 12 VOCs that are negatively correlated with cytokines load onto PC1 > 0 where the mice with the lowest total cytokines cluster, and the remaining 24 positively-correlated VOCs load onto PC1 < 0 with the mice with moderate-to-high levels of cytokines. Additional information about the VOCs, including putative identities, is provided in Supplementary Table S3.

When we performed PCA using only the 36 immune-correlated VOCs as variables, we observed that twice as much variance is explained by PC1 compared to the PCA with all 91 VOCs (Figure 4a versus Supplementary Figure S4), and we observed that the separation of mice based on their BALF VOC profiles follows the pattern of total cytokine production on PC1 (Figure 4a). On PC2, we observed that the mice with the highest cytokine levels have VOC profiles that differ by the infecting strain, separating the RS and Silveira mice into different quadrants. However, the VOC profiles of five mice with the lowest cytokine levels are not separated by fungal strain, though they are separated from the control mice. Evaluating the loadings of the VOCs on PC1, which describes the importance of each VOC on separating the mice along PC1 (Figure 4b), we observed that the loadings mirror the Kendall correlations between VOCs and cytokines: the 12 VOCs that are negatively correlated with cytokines load onto PC1 > 0 where the mice with the lowest total cytokines are clustered, and the remaining 24 positively-correlated VOCs load onto PC1 < 0 with the mice with moderate-to-high levels of cytokines. On PC2, four volatiles (21, 60, 68, and 69) separate the control mice from the five Cocci-inoculated mice with the lowest cytokines (Figure 4b). Additionally, the mice with moderate-to-high cytokines separate on PC2 based on the strain with which they were infected.

**Figure 5:**
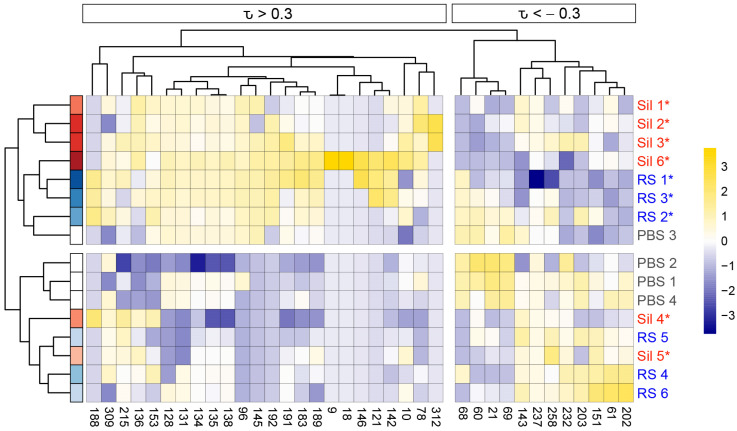
Immune-correlated VOCs cluster mice by total cytokine levels in BALF. Hierarchical clustering analysis (HCA) of 12 Cocci-inoculated and 4 PBS mice (rows) based on the relative abundance of 36 immune-correlated VOCs (columns) shows the mice are separated into two main clusters that reflect total BALF cytokine production. Additionally, the VOCs are divided into two clusters, those that are positively correlated (τ > 0.3) with cytokine production and those that are negatively correlated (τ < −0.3). Clustering of mice and VOCs used Pearson correlations with average linkage. Mice are color-coded by strain (blue = *C. immitis* RS; red = *C. posadasii* Sil) and a color gradient indicating total cytokine abundance, with darker color meaning higher abundance; disseminated disease is indicated with an asterisk (fungal counts in the spleen and brain are provided in Supplementary Table S2). Kendall correlation between volatiles and cytokines is noted above the dendrogram, with τ > 0.3 for positive correlations and τ < −0.3 for negative. Volatiles abundances (mean-centered and scaled to unit variance) are represented in the heat map. Additional information about the VOCs, including putative identities, is provided in Supplementary Table S3.

HCA using the 36 immune-correlated VOCs as variables recapitulated key features of the PCA, with mice separating into two main clusters based on cytokine production, and the mice with moderate-to-high cytokine production clustering by strain of infection (Figure 5). Additionally, the 36 volatiles divided into two clusters: volatiles that are positively correlated (τ > 0.3) with cytokine production in one cluster, and the negatively correlated (τ < −0.3) volatiles in another. This division is also what drives the variance across PC1 (Figure 4b). Six of the 36 immune-correlated VOCs are uniquely produced by mice with moderate-to-high cytokine production (VOCs 9, 18, 121, 146, 192, and 312; Figure 5 and Supplemental Table S3), with three of them only detectable in the BALF of Sil 6 and RS 1, who had the most severe disease.

## 4. Discussion

For this study, we sought to characterize the volatilome of a murine model of coccidioidomycosis lung infection as a proof of concept for developing a breath test for Valley fever. We examined the BALF of mice infected with *C. immitis* strain RS and *C. posadasii* strain Silveira as well as sham-infected controls inoculated with PBS. We initially anticipated that all Cocci-inoculated mice would have comparable cytokine levels in response to infection, yielding two distinctive groups: infected and uninfected. However, what we found was a gradient of cytokine abundance across the mice, with RS 5 and RS 6 producing total cytokine concentrations similar to the PBS controls (Figure 1), though we can see that even these mice have a different cytokine profile than that of the PBS mice (Figure S2 and Figure 2), indicating that there was an immune response to fungal exposure. While we strove to give each mouse the same dose of arthroconidia, due to the intranasal route of administration, it is possible that some mice swallowed a portion of the inoculum, reducing the overall dose to the lung and lowering the immune response [27,28]. Though inconsistencies in dosing are expected to be the main driver of the variations we observed in the cytokine levels, we do not rule out the possibility that interindividual differences in the hosts may also play a role. While C57BL/6J mice are inbred, other lung infection studies with this mouse strain have observed differences between individual mice given the same intranasal dose of a pathogen. Zhu et al. quantified the bacterial cell counts of homogenized lung tissue at multiple time points after 72 C57BL/6J mice were infected with either *Pseudomonas aeruginosa* or *Staphylococcus aureus* [8]. Six mice were killed at each time point, and while the mice had very similar bacterial cell counts for the first four time points postinoculation (i.e., CFU/lung within one order of magnitude, up to 48 h postinoculation [p.i.]), by 72 h and 120 h p.i. differences in the response to infection emerged; some mice had cleared the infection (i.e., no viable bacteria recovered), some had bacterial cell counts in their lungs ranging from 10^3^ to 10^8^, and a few mice succumbed to their infections. Franchina et al. observed a 100-fold difference in bacterial cell counts in the BALF of C57BL/6J mice infected with *Mycobacterium tuberculosis* at either 24 or 48 h postinfection [13]. Additionally, they observed a 100,000-fold difference in the number of neutrophils and macrophages present in the BALF of mice at each time point. Therefore, the variability in BALF cytokines that we observed within the RS- and Sil-inoculated cohorts are consistent with other murine pneumonia model studies in this mouse strain.

Of the 26 named compounds that we detected in the BALF of the infected mice, only one, decanal, had previously been detected and identified in in vitro cultures of *Coccidioides* spp. [32], though additional VOCs from the 65 unnamed compounds may also have been produced by the fungus in vitro. The relative lack of in vitro volatiles translating into this present study is not surprising, given the changes that *Coccidioides* undergoes within a mammalian host [58] and the emergent volatilome that arises via host–microbe interactions [10]. A prior mouse model study and two human studies on ventilator-associated pneumonia reported that only about a quarter to a third of bacterial in vitro volatiles are also detected in the breath of the infected host [7,46,59]. Further, the host makes significant contributions to the infection volatilome. In a murine lung infection model, Bean et al. showed that the breathprints of mice are altered for up to 120 h postinoculation upon exposure to bacterial cell lysates in a manner that was both bacterial species-specific and correlated with the immune response [10]. In humans, breath VOCs are altered after vaccination, as demonstrated in a study of persons receiving live attenuated influenza vaccine [60]. In our data, more than half of the BALF volatiles that we detected are known to be produced by the host. Fifty-three of the volatiles were in the BALF of all four of the control mice, and of these, 19 were correlated with the cytokines in infected mice (Supplementary Table S2), suggesting that the abundance of these host VOCs are altered in response to infection. Thus, given the significant contributions that the host makes to infection volatiles, the most direct route to the development of breath biomarkers for Valley fever will be in human clinical studies.

In the Cocci-inoculated mice with higher cytokine concentrations in BALF, we observed that the immune-correlated VOCs separate the mice by the strain of infection (Figure 4 and Figure 5). Because this study only included one strain from each species, we are unable to extrapolate the strain-specific separation of the infection volatiles that we observed here to the likelihood that there are inherent species-specific differences between volatiles from Valley fever caused by *C. immitis* vs. *C. posadasii* infection. However, our prior analysis of the in vitro volatilome of six strains each of *C. immitis* and *C. posadasii*, which included strains RS and Silveira, suggest that it is unlikely that we would observe interspecies differences in infection volatiles because intraspecies volatilomes are highly diverse [32]. Additionally, while both RS and Silveira are the type strains for their respective species, they both have characteristics that make us cautious to generalize these results to other strains of *C. immitis* or *C. posadasii*. Recent whole-genome sequencing of RS revealed that it is a hybrid strain with approximately 20% of its genome from *C. posadasii* [61]. Further, our in vitro volatilome study suggests RS has a metabolism that differs from many other *Coccidioides* strains, especially in the mycelial form, where it failed to produce many key volatile compounds that differentiate that life-form from spherules [32]. Silveira is often selected in vaccine challenges due to its high virulence in mice, which may not be representative of all *C. posadasii*. Previous *Coccidioides* mouse studies have also shown a variation in immune response based on the mouse strains [62,63], which compounds the challenge of extrapolating the differences we observed between the volatilomes of RS and Silveira-inoculated mice in this proof-of-concept study. To understand the variability of Valley fever infection volatiles and the impact that may have on the development of a Valley fever breath test, additional *Coccidioides* strains and murine strains need to be included in future murine model infection studies.

This proof-of-concept study shows that lung volatiles correlate with cytokine concentrations, and thus it may be possible to develop a Valley fever breath test that is sensitive enough to indicate disease severity in primary pulmonary coccidioidomycosis. While there was less distinction in the VOC profiles among the mice with low-to-moderate levels of disease (Figure 4 and Figure 5), this may be due to the limits of detection for VOCs from only 200 μL of BALF. By collecting higher BALF volumes or sampling and concentrating multiple breaths, we expect that we would be able to discriminate lower levels of Valley fever disease using lung VOCs. Clinically, a Valley fever breath test that also quantifies the severity of primary pulmonary disease would provide significant clinical benefit. It is recommended that patients with mild primary pulmonary coccidioidomycosis are initially monitored rather than treated with antifungals, as most patients resolve their infection without intervention [3]. However, disease severity is not strictly defined, and where a patient falls on the spectrum of mild-to-moderate disease is based upon signs and symptoms and the expert opinion of the clinician [64]. Therefore, the ideal Valley fever diagnostic will be able to distinguish not only the presence or absence of *Coccidioides* but also the level of infection, thereby limiting unnecessary antifungal treatment for individuals with self-limiting disease and reserving antifungals for the patients who will not be able to clear the infection on their own. It will be important going forward to not only define the breath biomarker threshold for initiating treatment but also to recognize this prescribing threshold will require a precision medicine approach. Mild coccidioidomycosis in patients who have comorbidities that increase their risk of prolonged infection (e.g., TNF inhibitors, chemotherapy, solid organ transplant) may necessitate the use of antifungal medications [2].

## 5. Conclusions

C57BL/6J mice inoculated with *C. immitis* RS or *C. posadasii* Silveira produced a gradient of cytokine responses to fungal exposure. We identified 36 VOCs in bronchoalveolar lavage fluid that were significantly correlated with cytokine production. These 36 VOCs clustered mice by total cytokine production and were also able to separate mice with moderate-to-high cytokine concentrations by *Coccidioides* strain. The data presented in this proof-of-concept study show that *Coccidioides* and/or the host produce volatile metabolites that may yield biomarkers for a Valley fever breath test that can detect the fungus and provide clinically relevant information on primary pulmonary disease severity. Additional studies with larger cohorts of mice and additional strains of *Coccidioides* are warranted in order to confirm and expand upon these findings.

## Figures and Tables

**Table 1:** Named volatiles detected in the BALF of *Coccidioides*-infected mice and previous reports in other fungal taxa.

VOC	Compound	Fungal Taxa *									References
		1	2	3	4	5	6	7	8	9	
10	isopropyl alcohol							x		x	[33,34,35]
18	2-methylpentane									x	[36]
21	2-methyl-2-propanol										
34	2-methyl-1-pentene										
37	hexane			x						x	[36,37]
54	2-ethoxy-2-methylpropane										
60	2-butanone		x	x				x		x	[34,36,38,39,40,41,42]
70	benzene		x								[43]
74	2-pentanone		x	x	x	x		x		x	[34,35,37,44,45]
83	1,3,5-cycloheptatriene										
93	cyclopentanone		x					x			[38,42]
99	p-xylene		x	x		x	x	x		x	[37,38,45,46,47,48]
104	1,3,5,7-cyclooctatetraene									x	[49]
121	2,2-dimethyl-octane										
123	benzaldehyde		x	x		x		x	x	x	[36,39,40,45,46,50,51]
129	octanal		x	x						x	[39,46,48,52]
136	2-ethyl-1-hexanol		x	x		x	x	x		x	[38,39,44,53]
142	undecane		x			x		x		x	[34,35,38,43,45,47,52,54]
153	nonanal		x	x		x		x		x	[35,36,39,45,46,48,52,53,54,55]
159	(E)-4-dodecene										
160	dodecane					x				x	[45,53]
171	2,6-xylidine										
173	decanal	x		x		x		x		x	[32,35,36,39,45,46,53,55,56]
188	2,5-dimethyl-benzaldehyde			x							[39,46]
270	2,4-di-tert-butylphenol									x	[57]
307	diethyl phthalate					x				x	[45,54,56]

* Fungal taxa: 1 = *Coccidioides* spp.; 2 = *Aspergillus* spp.; 3 = *Candida* spp.; 4 = *Cladosporium cladosporoides*; 5 = *Fusarium* spp.; 6 = *Mucor* spp.; 7 = *Penicillium* spp.; 8 = *Cyberlindnera jadinii*; 9 = Other spp., including environmental fungal species, *Alternaria brassicae*, *Botrytis cinerea*, *Periconia* spp., *Phytophthora infestans*, *Saccharomyces cerevisiae*, *Tricoderma* spp.

## Data Availability

Metabolomic data (chemical feature peak areas and retention time information) included in this study are available at the NIH Common Fund’s National Metabolomics Data Repository (NMDR) website, the Metabolomics Workbench, at www.metabolomicsworkbench.org, assigned project ID PR0001064 and study ID ST002350 (https://dx.doi.org/10.21228/M85H6W).

## Supplementary Materials

The following supporting information can be downloaded at: https://www.mdpi.com/article/10.3390/jof9010115/s1. Table S1: Parameters for HS-SPME and GC×GC-TOFMS analysis, and data processing and alignment, Figure S1: Data postprocessing workflow, Table S2: Table of fungal dissemination to mouse spleen and brain (CFU/mL) and cytokine abundance (pg/mL), Figure S2: Log_2_ fold change of cytokine abundance of Cocci-inoculated mice relative to the mean cytokine abundance of the PBS-inoculated mice, Figure S3: Hierarchical clustering analysis (HCA) of 12 cocci-inoculated and 4 PBS-inoculated mice based on the relative abundance of 26 cytokines, Table S3: Table of 91 *Coccidioides* VOCs detected in the headspace of mouse bronchoalveolar lavage fluid samples, Figure S4: Principal component analysis (PCA) score plot using 91 volatile organic compounds (VOCs) detected in the BALF of Cocci- and PBS-inoculated mice, Figure S5: Kendall correlation plot of the abundance of 26 cytokines and 91 volatile organic compounds (VOCs) in 12 cocci-inoculated and 4 PBS mice.
