# Supplementary material for: Volatile Metabolites in Lavage Fluid Are Correlated with Cytokine Production in a Valley Fever Murine Model
Source: J Fungi (Basel). 2023 Jan 14;9(1):115. doi: 10.3390/jof9010115 (PMC9864585; doi:10.3390/jof9010115)
Supplement: Supplementary file 1 [file jof-09-00115-s001.zip › Higgins_Keppler_Supplement_Final.pdf]

Supplementary Information for:

Volatile metabolites in lavage fluid are correlated with cytokine production in a Valley fever murine model

Emily A. Higgins Keppler [1,2], Marley C. Caballero Van Dyke [3,†], Heather L. Mead [3,‡], Douglas F. Lake [1], D. Mitchell Magee [4], Bridget M. Barker [3] and Heather D. Bean [1,2,*]

[1] School of Life Sciences, Arizona State University, Tempe, AZ 85287, USA; ehiggins@asu.edu (E.A.H.K.); douglas.lake@asu.edu (D.F.L.)
[2] Center for Fundamental and Applied Microbiomics, The Biodesign Institute, Arizona State University, Tempe, AZ 85287, USA
[3] Pathogen and Microbiome Institute, Northern Arizona University, Flagstaff, AZ 86011, USA; marley.vandyke@utsouthwestern.edu (M.C.C.V.D.); hmead@tgen.org (H.L.M.); bridget.barker@nau.edu (B.M.B.)
[4] Center for Personalized Diagnostics, The Biodesign Institute, Arizona State University, Tempe, AZ 85287, USA; mitch.magee@asu.edu
* Correspondence: heather.d.bean@asu.edu
† Current address: Department of Microbiology, University of Texas Southwestern Medical Center,
Dallas, TX 75390, USA.
‡ Current address: The Translational Genomics Research Institute (TGen), Flagstaff, AZ 86005, USA.

Methods

Table S1: Parameters for HS-SPME and GC×GC-TOFMS analysis, and data processing and alignment

**Autosampler Method**	
Instrument description	Gerstel® MPS Pro®
Software description	Gerstel® Maestro® (version 1.5.3.2)
**Sampling Parameters**	
Cooled tray temperature	4 °C
Solid-phase microextraction (SPME)	Manufacturer: Supelco®<br>Fiber type: PDMS/CAR/DVB (2 cm; 50/30 μm)
Incubation time	2 min
Agitator parameters, incubation	Temperature: 50 °C<br>On time: 10 s<br>Off time: 1 s<br>Speed: 600 rpm
Agitation, sampling	On
Vial penetration	21 mm
Extraction time	10 min
Injection penetration	67 mm
Desorption time	180 s
**Inlet (CIS) Parameters**	
Initial temperature	250 °C
Equilibrium time	0.05 min
Initial time	0.10 min
Ramp rate	12 $°C \cdot s^{-1}$
End temperature	250 °C
Hold time	10.5 min

**GC×GC Method**	
Instrument description	Agilent® 7890B
Column configuration	Column 1: Rxi®-624Sil MS, 60 m × 0.25 mm × 1.4 *μm*<br>Column 2: Stabilwax®, 1 m × 0.25 mm × 0.5 *μm*
Carrier gas	Helium, 2 $mL \cdot min^{-1}$ (constant)
Front inlet type	Gerstel®
Front inlet mode	Splitless
Front inlet septum purge flow	1 $mL \cdot min^{-1}$
Front inlet septum purge time	300 s
Front inlet purge flow	50 $mL \cdot min^{-1}$
Front inlet total purge flow	52 $mL \cdot min^{-1}$
Oven equilibration time	5 s
Primary oven temperature ramp	Initial temperature: 35 °C<br>Initial time: 0.5 min

	Ramp rate: 5 C·min^{-1}<br>Final temperature: 230 °C<br>Hold time: 5 min
Secondary oven temperature offset	+5 °C (relative to primary oven)
Modulator temperature offset	+15 °C (relative to secondary oven)
Modulation timing	Modulation period: 2.00 s<br>Hot pulse time: 0.50 s<br>Cold pulse time: 0.50 s
Transfer line temperature	250 °C

Mass Spectrometry Method	
Instrument description	LECO® Pegasus® 4D
Use GC method total time for MS method total time	Yes
Acquisition delay	180 s
Filament active time	180 s to end of run
Start mass/End mass	35/400
Acquisition rate	100 spectra·s^{-1}
Optimized voltage offset	+50 V
Electron energy	-70 eV
Ion source temperature	250 °C

Data Processing Method	
Software description	LECO® ChromaTOF® and Statistical Compare (version 4.71.0.0)
Baseline tracking/Offset	Entire run/0.5 (through middle of noise)
Data points averaged for smoothing	Auto
First dimension peak width	12 slices
Mass spectral match required to combine peaks	600
Second dimension peak width	0.15
Min. subpeak signal-to-noise (S/N) for retention	6
Integration approach	Traditional
Peak finding	S/N: 50<br>Number of apexing masses: 2
Mass spec libraries for searching	NIST® 2014
Mass to use for area/height calculation	Unique mass
Alignment analyte match criteria	Spectral match mass threshold: 10<br>Minimum spectral similarity match: 600<br>Max. number of modulation periods apart: 1<br>Max. retention time difference (s): 0.1<br>S/N for second peak find: 5
Criteria for inclusion of analytes	Min. number of samples that contain analyte: 1

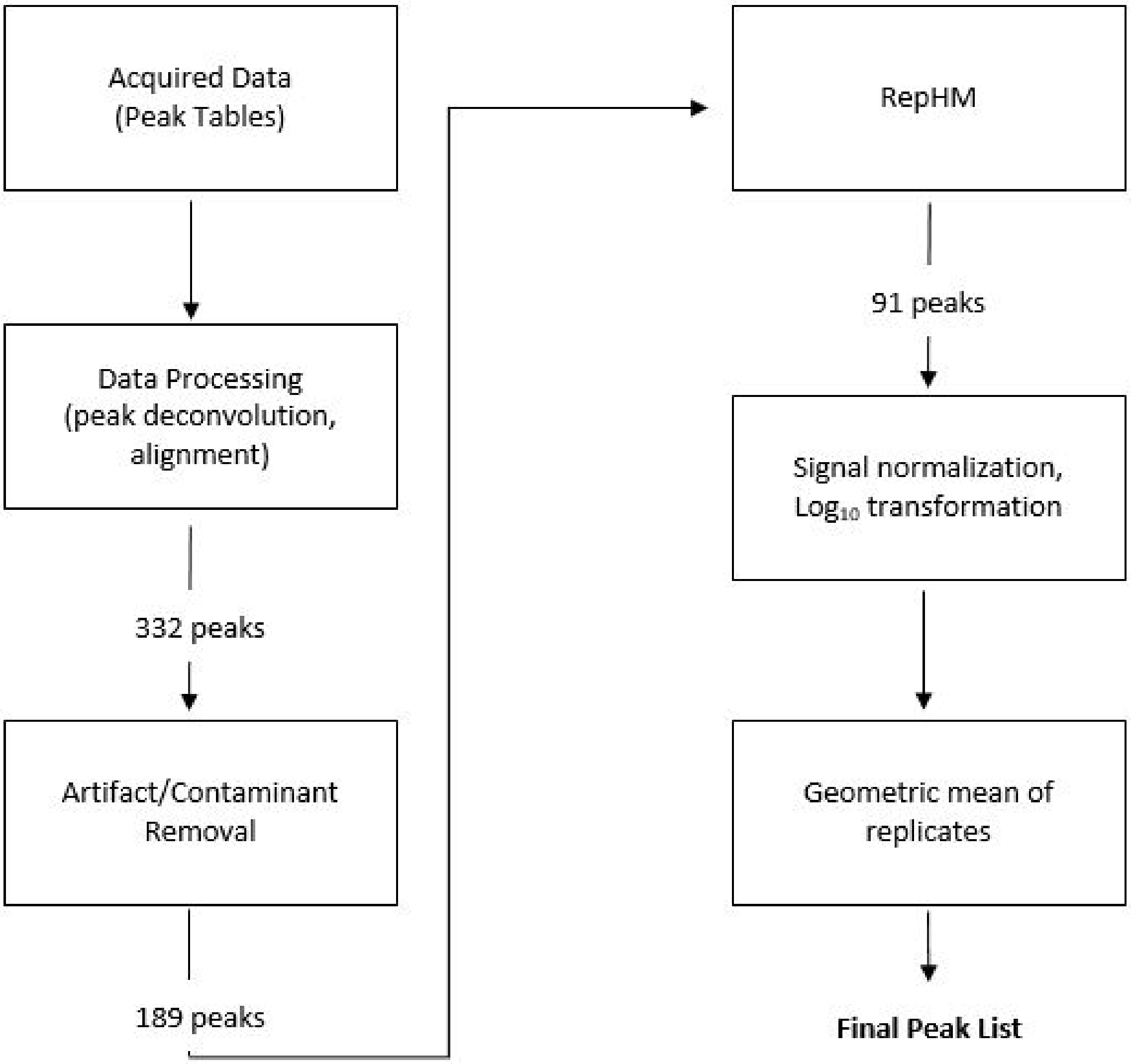

Figure S1: Data post processing workflow

Results

Table S2: Table of fungal dissemination to mouse spleen and brain (CFU/ml) and cytokine abundance (pg/ml).

	Dissemination (CFU/ml)		Anti-Inflammatory (pg/ml)					Pro-inflammatory (pg/ml)									
**Mouse**	**Spleen**	**Brain**	**IL-4**	**IL-5**	**IL-10**	**IL-13**	**IL-22**	**IL-1β**	**IL-2**	**IL-6**	**IL-9**	**IL-12p70**	**IL-17**	**IL-18**	**IL-23**	**TNF-α**	**IFN-γ**
**PBS 1**	0	0	9	6	18	35	7	6	21	10	15	14	9	11	16	17	14
**PBS 2**	0	0	8	5	21	33	7	5	26	10	17	12	9	11	25	26	20
**PBS 3**	0	0	9	6	21	30	7	6	25	13	17	14	11	11	19	19	18
**PBS 4**	0	0	9	6	20	35	7	6	23	11	19	15	10	11	19	20	18
**RS 1**	10	8	95	64	157	108	44	49	47	6640	44	233	264	436	60	537	78
**RS 2**	160	1	80	34	74	63	20	21	29	2734	27	104	108	214	35	160	44
**RS 3**	16	9	76	47	105	72	25	27	37	3828	33	140	157	268	47	238	56
**RS 4**	0	0	29	13	31	50	10	10	26	836	21	39	44	67	25	57	30
**RS 5**	0	0	9	6	14	33	7	6	18	38	14	13	10	13	12	15	12
**RS 6**	0	0	11	6	18	30	7	6	20	16	16	14	10	12	14	16	18
**Sil 1**	31	7	128	64	41	57	17	16	27	1259	25	59	77	140	21	73	27
**Sil 2**	31	0	259	88	34	67	16	16	27	1122	27	54	55	170	18	67	18
**Sil 3**	620	3	213	112	42	62	23	17	27	1309	27	66	55	171	23	114	42
**Sil 4**	13	0	72	20	32	48	11	12	27	1045	20	48	51	100	21	65	24
**Sil 5**	30	7	28	10	20	33	8	7	22	238	18	25	20	34	21	32	22
**Sil 6**	9400	8	329	333	83	91	44	33	43	2978	39	129	240	316	35	278	50

	Multi-faceted (pg/ml)		Chemokine (pg/ml)								
**Mouse**	**IL-27**	**Gm-CSF**	**Eotaxin**	**IP-10**	**KC**	**MIP1α**	**MIP1β**	**MIP2**	**MCP-1**	**MCP-3**	**RANTES**
**PBS 1**	8	8	19	22	12	47	23	9	13	20	57
**PBS 2**	7	7	19	24	10	46	18	9	12	20	45
**PBS 3**	7	8	20	25	11	45	18	9	12	20	51
**PBS 4**	7	8	24	24	11	45	23	9	13	21	60
**RS 1**	26	206	212	130	422	2944	1037	1892	110	1931	96
**RS 2**	15	96	118	98	210	722	279	249	35	738	77
**RS 3**	19	116	134	140	283	1595	600	438	44	1022	71
**RS 4**	10	26	39	46	108	560	167	61	18	225	56
**RS 5**	7	8	23	25	15	75	25	11	12	36	51
**RS 6**	7	8	22	70	13	55	22	11	13	126	47
**Sil 1**	11	46	179	154	64	504	365	21	37	3101	63
**Sil 2**	13	46	168	599	73	689	495	27	66	6295	80
**Sil 3**	14	49	178	376	73	950	540	44	72	5602	69
**Sil 4**	10	35	107	93	113	600	280	23	23	1553	59
**Sil 5**	8	14	51	50	37	190	87	13	15	200	48
**Sil 6**	20	107	687	826	163	2489	1897	118	135	5758	92

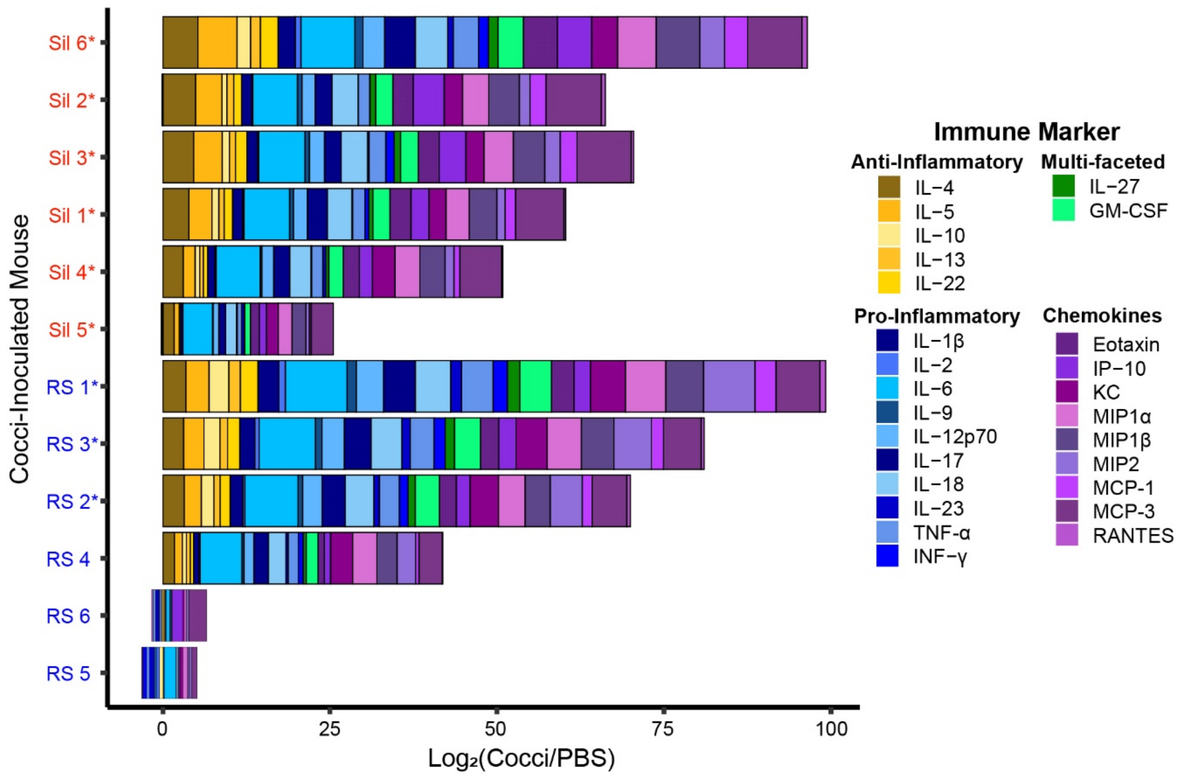

Figure S2: The cytokine profiles in *Coccidioides*-inoculated mice differ from PBS controls. Log_2 fold change of cytokine abundances of Cocci-inoculated mice (*C. immitis* strain RS (blue) and *C. posadasii* strain Silveira (Sil; red)) relative to the mean cytokine abundances of the PBS-inoculated mice. Mice with disseminated disease are indicated with an asterisk (*; Fungal counts in the spleen and brain are provided in Supplementary Table S2). Immune markers are color-coded by type and should be read from left to right in the bar graph in order to match them with their labels in the legend, listed from top to bottom.

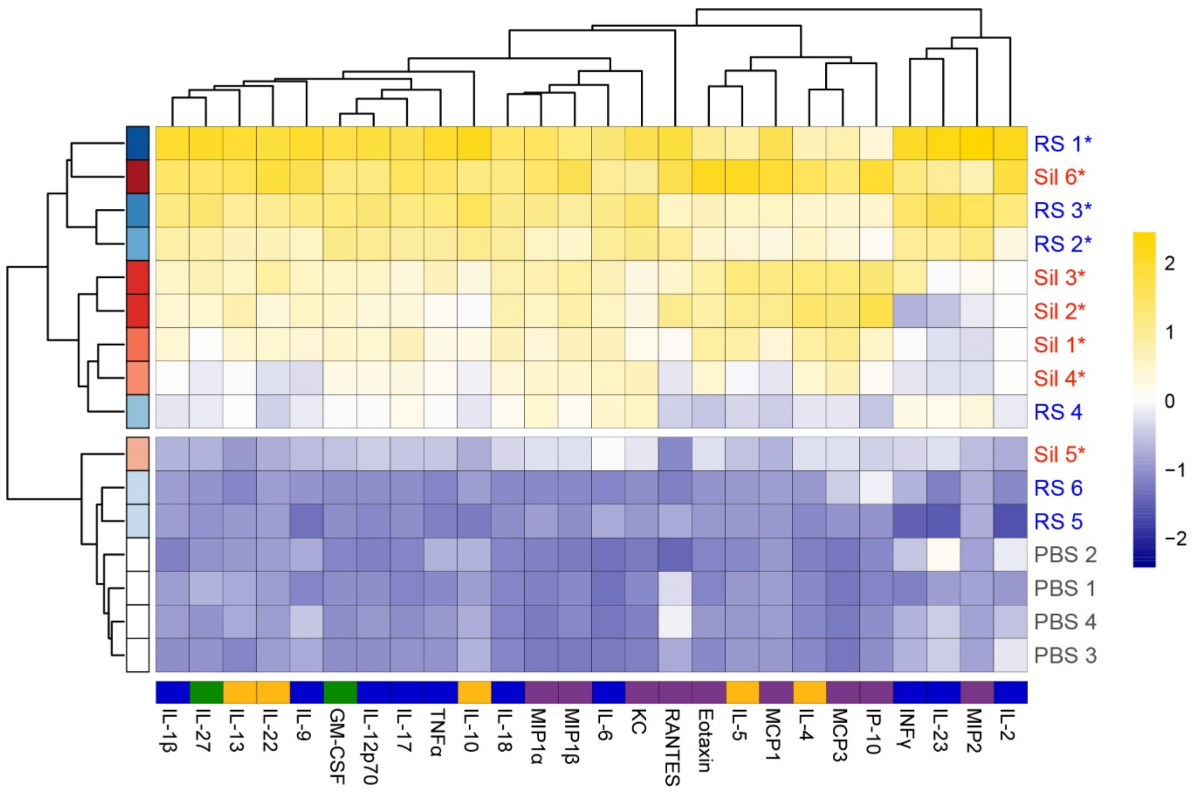

Figure S3: Mice cluster by their total cytokine abundance, not by *Coccidioides* strain. Hierarchical clustering analysis (HCA) of 12 Cocci-inoculated and 4 PBS-inoculated mice (rows) based on the relative abundances of 26 cytokines (columns). Clustering of mice and cytokines uses Euclidian distance with average linkage. Mice are color-coded by strain (blue = *C. immitis* RS; red = *C. posadasii* Sil) and a color gradient indicating total cytokine abundance, with darker color meaning higher abundance; disseminated disease is indicated with an asterisk (*; Fungal counts in the spleen and brain are provided in Supplementary Table S2). Cytokines are color-coded by type (anti-inflammatory in yellow, pro-inflammatory in blue, multi-faceted in green, and chemokines in purple), and their abundances (mean-centered and scaled to unit variance) are represented in the heat map.

Table S3: See **Supplementary Excel File**. Table of 91 *Coccidioides* VOCs detected in the headspace of mouse bronchoalveolar lavage fluid samples.

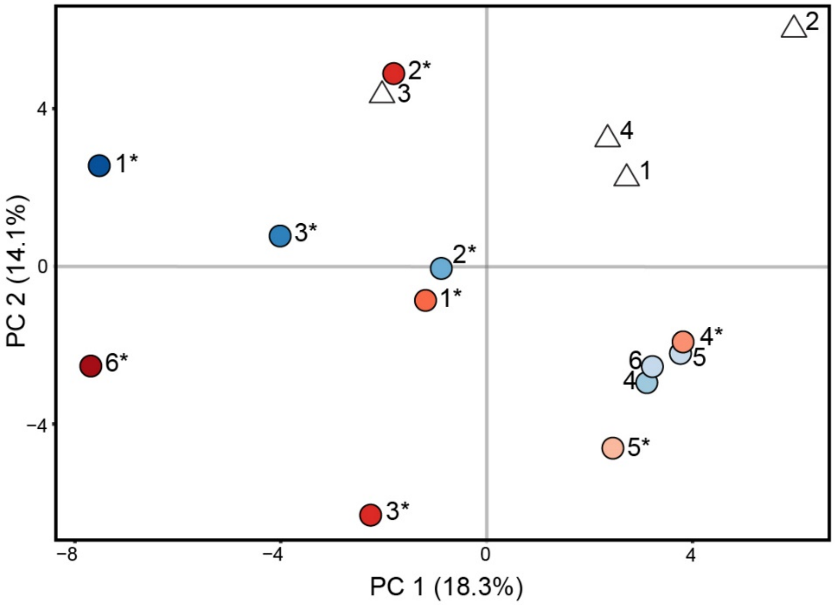

Figure S4: The separation of mice using the total BALF volatilome suggests a relationship between volatile profiles and total cytokine abundance. A principal component analysis (PCA) score plot of 16 mice inoculated with C. immitis RS (blue circles, n=6), C. posadasii Silveira (red circles, n=6) or PBS (white triangles, n=4) as observations, using 91 BALF VOCs as variables shows the mice separate on PC 1 in a pattern that reflects total cytokine abundance. The color gradient in the observation markers indicates total cytokine abundance, with the darkest colors indicating the highest abundances; mice with disseminated disease are indicated with an asterisk (*).

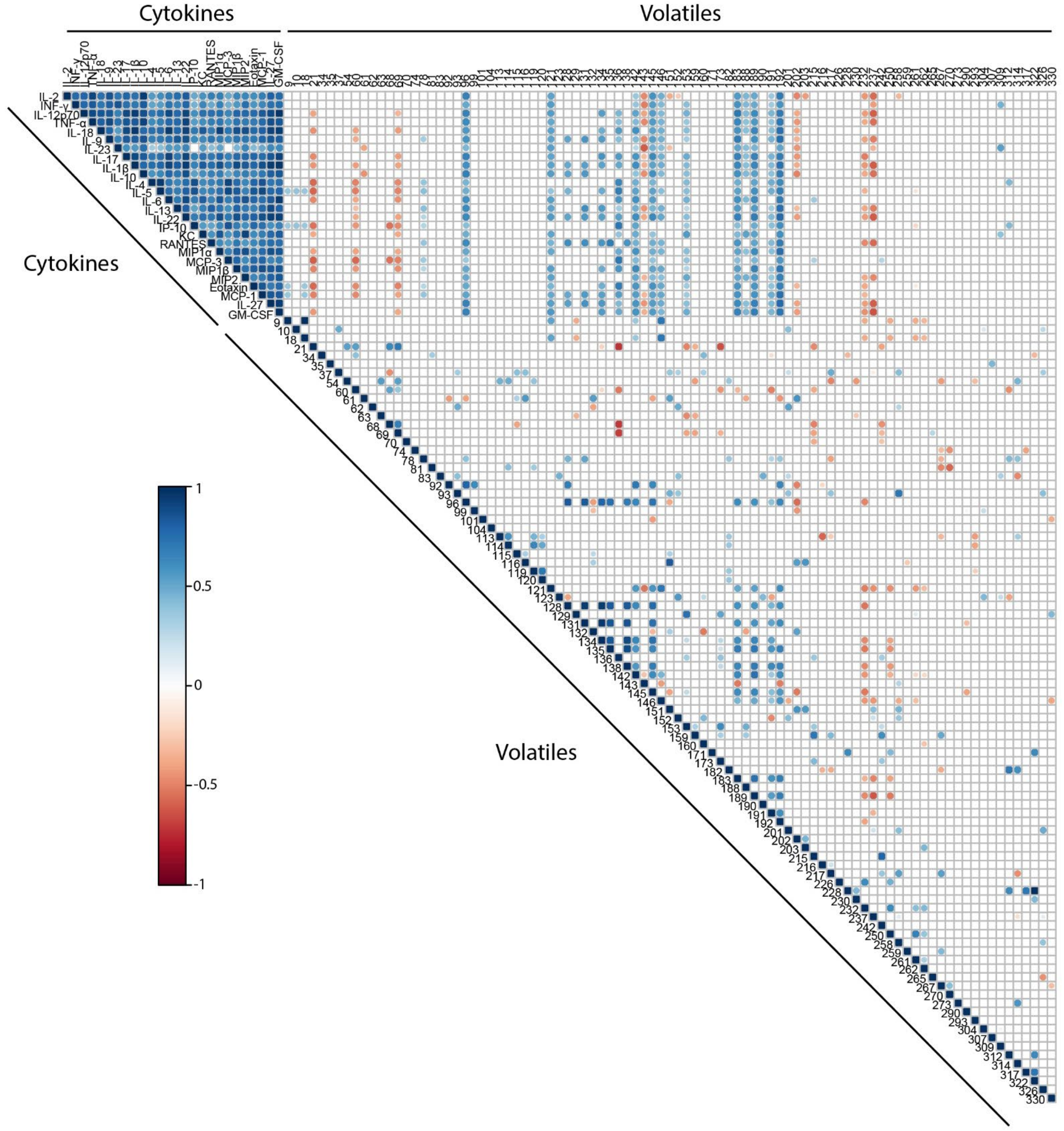

Figure S5: A subset of the 91 VOCs in BALF are correlated to cytokines. Kendall correlations were calculated between 26 cytokines and 91 volatile organic compounds (VOCs) in the BALF of 12 Cocci-inoculated and 4 PBS mice. Circles indicate statistically significant correlations ($P < 0.05$), with positive correlations in blue ($\tau > 0.3$), negative correlations in red ($\tau < -0.3$), and darker colors and larger sizes indicating a stronger correlation.
